# P-cadherin and the journey to cancer metastasis

**DOI:** 10.1186/s12943-015-0448-4

**Published:** 2015-10-06

**Authors:** André Filipe Vieira, Joana Paredes

**Affiliations:** Instituto de Investigação e Inovação em Saúde, Universidade do Porto, Porto, Portugal; IPATIMUP – Instituto de Patologia e Imunologia Molecular da Universidade do Porto, Rua Júlio Amaral de Carvalho, N. 45, 4200-135 Porto, Portugal; Faculdade de Medicina da Universidade do Porto, Porto, Portugal

**Keywords:** P-cadherin, Metastasis, Cancer, Cancer stem cells, Invasion

## Abstract

P-cadherin is a classical cell-to-cell adhesion molecule with a homeostatic function in several normal tissues. However, its behaviour in the malignant setting is notably dependent on the cellular context. In some tumour models, such as melanoma and oral squamous cell carcinoma, P-cadherin acts as a tumour suppressor, since its absence is associated with a more aggressive cancer cell phenotype; nevertheless, the overexpression of this molecule is linked to significant tumour promoting effects in the breast, ovarian, prostate, endometrial, skin, gastric, pancreas and colon neoplasms. Herein, we review the role of P-cadherin in cancer cell invasion, as well as in loco-regional and distant metastatic dissemination. We focus in P-cadherin signalling pathways that are activated to induce invasion and metastasis, as well as cancer stem cell properties. The signalling network downstream of P-cadherin is notably dependent on the cellular and tissue context and includes the activation of integrin molecules, receptor tyrosine kinases, small molecule GTPases, EMT transcription factors, and crosstalk with other cadherin family members. As new oncogenic molecular pathways mediated by P-cadherin are uncovered, putative therapeutic options can be tested, which will allow for the targeting of invasion or metastatic disease, depending on the tumour model.

## Introduction

P-cadherin was described for the first time in 1986, as part of “a novel class of cadherins that appeared in developing mouse embryos” and its name is derived from the site where this adhesion molecule was firstly characterized, the placenta [1]. Embryonic histogenesis studies have shown that P-cadherin expression is confined to the extra-embryonic ectoderm and visceral endoderm, the structures originating the placenta in mice [2]. P-cadherin is a calcium dependent cell–cell adhesion glycoprotein, which has a crucial role in the conservation of the structural integrity of epithelial tissues. Similarly to other members of the cadherin family, P-cadherin regulates several cellular homeostatic processes that participate in embryonic development and maintain adult tissue architecture, being important for cell differentiation, cell shape, cell polarity, growth and migration [3–5]. Sharing about 67 % of homology with the E-cadherin protein, P-cadherin differs mainly in the extracellular portion and it is far less characterized [6, 7].

Despite being expressed in human foetal structures [6, 8], P-cadherin is present in several adult tissues, usually co-expressed with E-cadherin, such as the basal layer of the epidermis, the breast and the prostate, as well as the mesothelium, the ovary, the cervix, the hair follicle, and the corneal endothelium [1, 9]. Despite its role in the maintenance of tissue architecture [10, 11], P-cadherin is notably involved in disease states, namely in specific hereditary genetic disorders and in cancer.

## P-cadherin in physiology: role in stem cell biology, cell differentiation, and tissue architecture

P-cadherin contributes to the biology of stem cells of the normal mammary gland and the hair follicle, and its presence has been reported in embryonic stem cells [6, 11–13]. Mammary fractionation experiments in mice have shown that P-cadherin is found in a subpopulation of cells with a basal-like phenotype, co-expressed with SLUG, a transcription factor that regulates cadherin genes and the genes involved in mammary epithelial cell lineages [14]. In fact, P-cadherin expression is confined to the basal myoepithelial layer of the human mammary gland, eventually contributing to the suprabasal stem cell niche [15–17]. In the developing gland, P-cadherin is found in stem cells that give rise to the myoepithelial cell layer, termed cap cells [18, 19]. In contrast, in the mature lactating gland, P-cadherin is expressed in human alveolar cells, the units responsible for milk protein production [20]. In fact, a soluble form of P-cadherin corresponding to the extracellular domain is found in human breast milk [20]. Although the significance of this soluble P-cadherin fragment is not yet clarified, putative roles implicated in immunological response and cell signalling have been suggested.

In the growing hair follicle, P-cadherin is found in the small hair placodes, specifically in the early progenitor cells from hair germs, co-expressed with K14+ and α6-integrin/CD49f + markers, where it has a putative role in the differentiation of the growing hair follicle [13]. Furthermore, it was shown that P-cadherin is one of the cell surface proteins that allows the identification of the pluripotent population of human embryonic stem cells, being co-expressed with E-cadherin in this setting [12].

The function of P-cadherin has been significantly elucidated by P-cadherin inactivation studies performed in mice, as well as by data provided by P-cadherin germline mutations in humans. Radice and colleagues demonstrated that whole body deletion of P-cadherin disturbs normal mammopoiesis, as *CDH3*/P-cadherin-null female mice exhibit precocious mammary gland differentiation in the virgin state, with extensive alveolar differentiation. Moreover, these animals show increased risk of developing pre-neoplastic lesions, such as alveolar hyperplasia and ductal dysplasia with increasing age. These observations indicate that P-cadherin cell-cell interactions and signalling are important for limiting the growth of the mature luminal epithelial cells, being important for the maintenance of an undifferentiated state of the normal mammary gland, which points to the role of P-cadherin as a stem cell marker and active in modulating stem signalling pathways [21, 22].

Recent studies have clarified that P-cadherin expression is crucial to the maintenance of normal breast epithelial architecture. LaBarge’s group has used an antibody that specifically antagonizes P-cadherin cell-cell interactions in an *in vitro* self-organizing assay of the human mammary bilayer to show that the migration of mammary myoepithelial cells, occurring during normal sorting of both layers, was compromised [23]. Furthermore, using mammary cells isolated from P-cadherin knock-out mice, Andrew Ewald’s group has recently demonstrated that the loss of P-cadherin causes precocious branching morphogenesis in matrigel and enhanced sustained dissemination into collagen type I, pointing to the importance of this adhesion molecule in the maintenance of normal breast epithelial architecture [24]. It would be interesting to clarify the mechanisms behind the homeostatic function mediated by P-cadherin in the normal breast, since the loss of this adhesion molecule could cause the rupture of the myoepithelial cell layer and lead to pre-neoplastic lesions. Future studies at the cellular level should provide valuable information regarding the influence of P-cadherin in tissue architecture and cell shape, namely crosstalk with cell polarity determinants and other junctional proteins.

Although P-cadherin is not profusely expressed throughout the body, this molecule is also found in other sites besides the breast and hair follicle, namely in the basal layer of many adult tissues, where it is believed to act as a classical cell-cell adhesion molecule, possibly contributing to the undifferentiated state of epithelial cells. This is the case for skin, prostate and testis, pancreas, several organs of the digestive tract and urinary tract, lung and endometrium. P-cadherin is completely absent from heart muscle and brain [1, 9, 25].

In humans, loss of P-cadherin induces characteristic genetic syndromes. Several *CDH3*/P-cadherin germline mutations have been shown to cause P-cadherin functional inactivation, leading to developmental defects associated with hypotrichosis with juvenile macular dystrophy (HJMD) [26–28] and ectodermal dysplasia, ectrodactyly, and macular dystrophy (EEM syndrome) [29, 30]. Sixteen *CDH3* mutations have been associated with HJMD, which is a rare recessive disorder, characterized by hair loss heralding progressive macular degeneration and early blindness in the second to third decade of life. These mutations mainly disturb the Ca^2+^ binding and the cadherin domain or result in the synthesis of a truncated form of P-cadherin or in the absence of P-cadherin expression [26–28, 31–37]. EEM is another P-cadherin developmental defect associated syndrome, which is also characterized by sparse hair and macular dystrophy of the retina as HJMD, with the additional finding of split hand/foot malformation [29]. Different degrees of absence of bone structures, as well as syndactyly, have been described, the hands often being more severely affected than the feet. Kjaer and colleagues first established the link between families with EEM and homozygous mutations in *CDH3*/P-cadherin gene in affected individuals and, up to now, three *CDH3* gene mutations have been shown to lead to EEM syndrome [29, 30].

## P-cadherin as a double edge sword: a tumour suppressor or a tumour-promoting molecule?

Concerning carcinogenesis, the effective role of P-cadherin remains an object of debate, since it can behave differently depending on the molecular context and tumour cell model studied (Table 1). In melanoma, non-small cell lung carcinoma, oral squamous cell carcinoma and hepatocarcinoma, P-cadherin has a similar tumour suppressive behaviour to E-cadherin. However, in some tumour models, such as bladder, prostate and colon carcinomas, opposing effects have been found for P-cadherin, with some studies pointing to an associated tumour suppressive effect [38–40] and others pointing to the induction of aggressive behaviour [39, 41, 42], with the differences observed being related to the cell model, the immunodetection method, and the different functions attributed to P-cadherin in the membrane or in the cytoplasm. The pathology studies presented in this review focus in the membranous expression of P-cadherin. However, one important study that highlights the crucial issue of P-cadherin expression in cytoplasm vs. membrane was published by Mandeville and colleagues that showed that, in bladder carcinomas, the patients with membrane expression of P-cadherin showed a longer cancer-specific survival than the patients with cytoplasmic relocation of P-cadherin [38].

**Table 1:** P-cadherin expression in primary tumours and its relevance in malignancy. The tumour promoting or tumour suppressive effects associated with P-cadherin expression is dependent on the cellular and tissue context

Malignancy	Expression in neoplastic tissue/cells in comparison to normal tissue/cells	Behaviour	References
Breast cancer	Up-regulation	Tumour promoting	Paredes et al., 2005 [53]
Gastric cancer	Up-regulation	Tumour promoting	Shimoyama et al., 1991 [54]
Endometrial cancer	Up-regulation	Tumour promoting	Moreno-Bueno et al., 2003 [55] Stefansson et al., 2004 [56]
Ovarian cancer	Up-regulation	Tumour promoting	Patel et al., 2003 [57]
Pancreatic cancer	Up-regulation	Tumour promoting	Taniuchi et al., 2005 [58]
Basocellular and squamous carcinoma of the skin	Up-regulation	Tumour promoting	Wakita et al.,1998 [59] Hardy et al., 2002 [60]
Cholangiocarcinoma	Up-regulation	Tumour promoting	Obama K et al., 2005 [111]
Colorectal carcinoma	Up-regulation	Tumour promoting	Hardy et al., 2002 [60] Imai et al., 2008 [9] Sun et al., 2011 [71]
	Down-regulation	Tumour suppressive	Van Marck et al., 2011 [39]
Bladder cancer	Up-regulation	Tumour promoting	Wang et al., 2014 [41]
	Down-regulation	Tumour suppressive	Mandeville et al., 2008 [38] Van Marck et al., 2011 [39]
Prostate cancer	Up-regulation	Tumour promoting	Arenas et al., 2000 [42]
	Down-regulation	Tumour suppressive	Jarrard et al., 1997 [40]
Melanoma	Down-regulation	Tumour suppressive	Fenouille et al., 2012 [92] Van Marck et al., 2005 [43] Jacobs et al., 2011 [44]
Non small cell lung cancer	Down-regulation	Tumour suppressive	Smythe et al., 1999 [45]
Oral squamous cell carcinoma	Down-regulation	Tumour suppressive	Lo Muzio et al., 2004 [47] Muñoz-Guerra et al., 2005 [48]
Hepatocellular carcinoma	Down-regulation	Tumour suppressive	Bauer R et al., 2014 [52]

Studies from Bracke and colleagues have shown an invasion suppressor role for E-cadherin and P-cadherin on melanoma, which decrease their membranous expression when the disease progresses to a metastatic stage [43, 44]. *De novo* expression of P-cadherin in melanoma cells reduced xenograft tumour growth and prolonged mouse survival in a model mimicking micrometastatic spread [44], as well as promoted adhesive cell-cell contacts and anti-invasive effects *in vitro*. This response was abolished when targeted mutations on the P-cadherin intracellular juxtamembrane domain or on its extracellular domain were introduced [43].

In lung carcinoma, a similar adhesive and tumour suppressive function was described for P-cadherin: in non-small cell carcinoma, a decrease in its expression was noted in 68 % of the patients [45]. Accordingly, the transfection of P-cadherin in the cellular model of Lewis lung carcinoma significantly increased cohesiveness of cancer cells and greatly reduced invasion [46].

Furthermore, absence of P-cadherin expression constitutes a hallmark of aggressive biological behaviour in oral squamous cell carcinoma (OSCC). P-cadherin was found to be downregulated in high grade OSCC and patients with tumours showing reduced or no P-cadherin membranous expression had poorer overall and disease-free survival rates than the group of P-cadherin-expressing tumours [47, 48]. Enhanced presenilin-1/γ-secretase expression in OSCC cell lines was found to lead to accumulation of P-cadherin in the cytoplasm and to reduced cell-cell adhesion of OSCC cells [49]. In OSCC cells, P-cadherin is able to interact with Wnt-related signalling molecules, namely the Robo-3/Slit-2 complex, being responsible for the inhibition of cellular migration [50]. Furthermore, it was shown that P-cadherin epithelial features in OSCC are triggered by GSK-3β-mediated inactivation and cytoplasmatic translocation of Snail [51].

In hepatocarcinoma, downregulation of P-cadherin was found to induce aggressive behaviour, being correlated to high-grade neoplastic lesions. Further, suppression of P-cadherin expression in hepatocarcinoma cells in culture increased cell proliferation [52].

In contrast to the models presented above, aberrant expression of P-cadherin associated with aggressive tumour behaviour is observed in breast [53], gastric [54], endometrial [55, 56], ovarian [57], prostate [42], pancreatic [58], bladder [41] and colorectal carcinomas [9], as well as in basocellular and squamous carcinomas of the skin [59]. Importantly, P-cadherin appears upregulated in the earlier stages of the malignant progress in most cases [60, 61]; however, the extent to which P-cadherin contributes to the carcinogenesis or the progression of these various tumours is still not clarified.

It is in breast cancer that this adhesion molecule has received more attention and the mechanisms leading to the major tumour promoting effects have been widely characterized. P-cadherin aberrant expression is associated with breast carcinomas of high histological grade, as well as with the expression of well established markers associated to poor patient prognosis, like Ki-67, EGFR, CK5, vimentin, p53 and HER-2, and negatively associated with the expression of hormonal receptors (ER and PgR) [53, 61–64]. In fact, P-cadherin overexpression is mainly found in the triple-negative and basal-like subgroup of breast cancers [65, 66] and it is strongly correlated with the presence of *BRCA1* mutations [67]. Interestingly, none of these reports showed a significant association with tumour size and lymph node metastasis.

## P-cadherin overexpression is associated with cancer cell invasion and metastatic dissemination

The relevance of P-cadherin in metastasis has been addressed by the analysis of clinicopathological data in primary tumours and matched lymph node metastasis, animal models of metastatic dissemination, and cell culture experiments using metastatic cellular models (Table 2).

**Table 2:** P-cadherin expression in invasion and metastasis and the corresponding signalling pathways. This table focuses on the major tumour promoting signalling effects mediated by P-cadherin in the metastatic setting

	Metastatic site	Phenotype	Signalling	Ref.
Breast cancer	Lymph nodes	Significant change of P-cad expression in the lymph nodes vs. primary tumour		Adamczyk et al., 2012 [69]
		Invasion, migration motility	Soluble P-cad, MMPs	Ribeiro et al., 2010 [75]
		Stem cell activity Adhesion to laminin	α6β 4-integrin, FAK, Src, Akt	Vieira et al., 2012 and 2014 [16, 17]
		Increased glycolysis and acid resistance	CA-IX, Glut-1	Sousa et al., 2014 [94]
Ovary	Peritoneum	P-cad necessary for adhesion of CaOV-3 cells to the peritoneum	P-cad/β 1-integrin	Ip et al., 2014 [85]
	Peritoneum	P-cad necessary for adhesion of CaOV-3 and OVCAR-3 (to ECM and peritoneal cells) P-cad inhibition attenuated tumour growth, ascites formation, and the number of metastatic implants	Gonadotropin-releasing hormone induces P-cadherin expression and α2, α5 and β1 integrin	Cheung et al., 2013 [70]
	Peritoneum	P-cad necessary for the activation of the IGF-1R by GnRH in CaOV-3 cells P-cad and p-IGF-1R coexpression was significantly stronger in metastasis compared with primary tumours	p120 phosphorylation by Gonadotropin-releasing hormone is dependent on P-cadherin and IGF-1R interaction	Cheung et al., 2011 [86]
Colon	Liver	Increased in liver metastasis of colon cancer, compared with the primary cancer tissue; Knock-down of P-cadherin in colon cancer cells (LOVO) resulted in developing fewer liver metastatic foci	P-cad inhibition in colon cancer cells (LOVO) induced the up-regulation of E-cadherin and the down-regulation of β-catenin and its downstream target molecules, including survivin and c-Myc.	Sun et al., 2011 [71]
Prostate cancer	Bone	P-cad expression in primary tumour associated with shorter time to skeletal metastasis		Gravdal et al., 2007 [72]
Gallbladder adenocarcinoma	Lymph nodes	P-cad expression in primary adenocarcinoma and squamous cell/adenosquamous carcinoma associated with lymph node metastasis		Yi et al. 2014 [68]

The association of P-cadherin expression in the primary tumour with lymph node involvement varies, with some reports pointing to a positive association, like in gallbladder adenocarcinoma [68], and others showing no significant association, such as in breast cancer [53]. Agnieszka and colleagues, by the use of a series of human breast carcinomas with matched lymph node metastasis, showed that P-cadherin expression varied considerably (increased or decreased) between primary tumour and nodal metastasis (29.2 % of total cases), and that all the primary cases with P-cadherin enhancement in the lymph nodes were triple negative breast carcinomas (i.e., breast carcinomas that are negative for ER, PR and HER2). Importantly, a low variation in staining intensity was found for ER, PR, HER2, CK5/6 and EGFR expression in the primary tumour and nodal metastasis (over 90 % of the cases showed the same level of expression for these markers), suggesting that P-cadherin may be a candidate prognostic biomarker to explore in the lymph nodes [69].

Using a model of human ovarian cancer metastasis, in which Caov-3 cells were intraperitoneally injected in nude mice, forming multiple secondary tumours on the mesenterium and small bowel, Cheung and colleagues showed that P-cadherin silencing leads to a significant decrease in the number of metastatic cells present in the ascitic fluid. The ascites volume and formation of tumour nodules were substantially decreased by β1 integrin and P-cadherin silencing, suggesting that these molecules might be involved in the adhesion of metastatic cells to the peritoneum [70].

In colon cancer, gene expression and immunohistochemistry analyses showed that P-cadherin expression was significantly higher in liver metastases than in paired primary colorectal cancer tumours. Knockdown of P-cadherin in colon cancer cells inhibited wound healing, proliferation, and colony formation and resulted in developing fewer liver metastatic foci *in vivo.* The effect of P-cadherin inhibition was associated with the down-regulation of β-catenin (βctn) and its downstream target molecules, including survivin and c-Myc [71].

P-cadherin expression in carcinomatous prostate is increased compared to normal prostate tissue [42] and this was significantly associated with bone metastasis [72]. Importantly, the migratory and invasive phenotype of prostate cancer cells was dependent on P-cadherin expression [73, 74].

In breast cancer, we found that overexpression of P-cadherin promotes cell motility, cell migration, as well as invasion capacity through matrigel [75]. A similar aggressive phenotype was also observed in bladder and pancreatic cancer cell lines [38, 39, 58]. Curiously, we have noticed that P-cadherin is able to induce invasion only in breast cancer cells that already express an endogenous and functional E-cadherin molecule [75–77]. In fact, breast carcinomas that co-express E- and P-cadherin show a patient survival that is worse than that associated with breast carcinomas that express only one of the cadherins or that do not express any of these adhesion molecules [58, 77].

A study by Sarrió and colleagues showed that when either P- or E- cadherin was expressed alone in a breast cell line that is negative for both cadherins, a similar gene expression pathway was activated, followed by the acquisition of a epithelial morphology and increased cell-cell adhesion. This suggests that P-cadherin *per se* is not enough to increase cancer aggressiveness in breast cancer and that when either P- or E-cadherin are expressed alone, both proteins act as suppressors of cancer properties [78]. However, this study did not analyse the cellular system where both E- and P-cadherin are co-expressed in the same cells, resembling the more frequent breast carcinomas in which aggressive invasion is observed when P- and E-cadherin are jointly expressed [79]. In this case, it has been proposed that the underlying mechanism leading to P-cadherin mediated tumour promoting properties comprises the perturbation of the tumour suppressive signalling mediated by E-cadherin [79].

The reports mentioned above clearly show the importance of studying the mechanisms mediated by P-cadherin that lead to the metastatic dissemination of cancer cells. These mechanisms may include changes in cadherin mechanotransduction, stem cell signalling, cadherin switching and epithelial to mesenchymal transition. Further, the tumour microenvironment and the crosstalk with non-tumour cells in the secondary site must also be considered.

## P-cadherin signalling pathways in invasion and metastasis

**Fig. 1:**
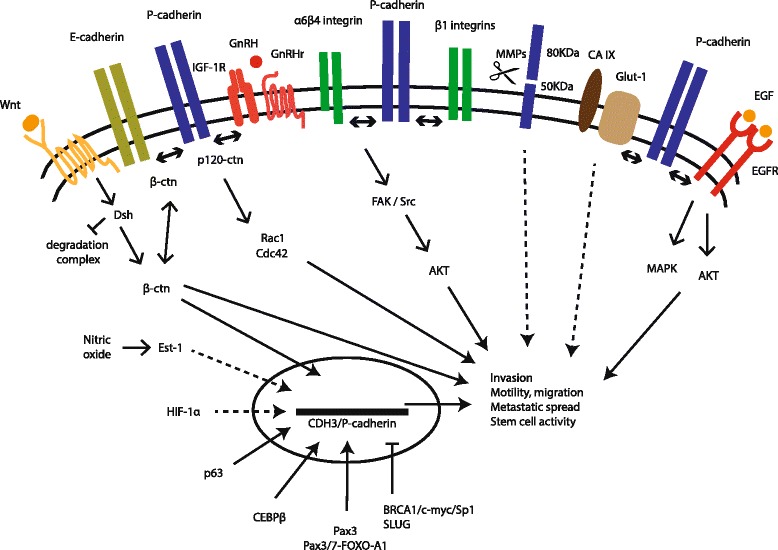
P-cadherin signalling pathways in the malignant setting. The tumour promoting effects mediated by P-cadherin include cell invasion, cell motility, stem cell activity and metastases formation in different tissue contexts (see text for details). (GnRH – Gonadotropin-releasing hormone; GnRHr - Gonadotropin-releasing hormone receptor; IGF-1R – Insulin-like growth factor receptor; MMP – matrix metalloproteinase; EGFR – epidermal growth factor receptor)

For the last two decades, most of the signalling described for P-cadherin has been derived from its oncogenic behaviour, with aberrantly elevated levels of P-cadherin playing a critical role in the augmentation of neoplastic signalling networks and in the further acquisition of aggressive tumour phenotypes (Fig. 1). In a classical perspective, P-cadherin mediates its intercellular signalling through the interaction with βctn and p120ctn molecules, which contribute to the stabilization of the adherens junction complex at the cell surface, allowing the physical interaction with the actin cytoskeleton. We have recently demonstrated that P-cadherin is able to interfere with the tumour suppressive function of E-cadherin in breast carcinomas and in cell culture, promoting cancer cell invasion by disrupting the interaction between E-cadherin and both p120ctn and βctn [79]. In fact, the breast carcinomas that co-express both adhesion molecules have a decrease in the membrane staining of p120-catenin (p120ctn) and an increase in the cytoplasmic localization for this protein [58, 77]. In pancreatic and ovarian cancer, it was shown that p120ctn, once in the cytoplasm, can activate Rho-GTPases, like Rac1 and Cdc42, altering the actin cytoskeleton polymerization and promoting cell motility [58, 80]. In the absence of E-cadherin expression, P-cadherin is able to suppress invasion by its strong interaction with catenins, in a similar way as E-cadherin in cell-cell adhesion [79]. Further, a recent report by Bazellieres and colleagues showed that both cadherins are able to compete for a joint mechanotransduction pathway in normal breast cells, showing that P- and E-cadherin play fundamentally distinct roles in controlling force transmission at intercellular junctions [81]. They suggest that P-cadherin is involved in increasing the levels of intercellular tension within a monolayer of MCF10A breast cells, whereas E-cadherin dictates the rate at which the intercellular tension builds up over time. The authors showed that in the absence of E-cadherin, P-cadherin is able to take over its role as a tension regulator, triggering mechanotransduction and preventing a decrease in intercellular tension, thus confirming at the mechanical level that where only P- or E-cadherin are expressed, these proteins act as suppressors of cancer invasion.

Transient or irreversible inactivation of the cadherin-catenin complexes by secreted factors, such as metalloproteinases (MMPs), produced by tumour and/or stromal cells can also occur. We have shown that P-cadherin overexpression promotes cell invasion, migration and motility accompanied by the secretion of MMP1 and MMP2, which lead to P-cadherin ectodomain cleavage, releasing the soluble P-cadherin fragment [75]. Further studies are needed to understand if MMPs activation by P-cadherin in breast cancer is exclusive for P-cadherin or this function can also be played by E-cadherin. Still, it is important to highlight that in this study the cellular context was E-cadherin positive.

Studies in breast and ovary carcinomas have demonstrated the connection between P-cadherin and the activation of integrin molecules, which gives cancer cells an advantage to attach to the underlying extracellular matrix. Our group has demonstrated an important interplay between P-cadherin and α6β4 integrin in breast cancer, which is linked with increased cancer cell adhesion to the basemement membrane substrate laminin. We showed that P-cadherin downstream signalling in response to laminin involves the activation of FAK, Src and AKT kinases, an association that was validated *in vivo* [16]. Although a direct link with metastatic dissemination was not established, the α6β4 integrin heterodimer was shown to be important for cell invasion, as well as for the maintenance of stem cell properties.

In the breast, the link of α6β4-integrin and P-cadherin is exclusive for P-cadherin and no such link was found for E-cadherin. However, in other contexts there seems to be crosstalk between α6β4-integrin and the classical E- and P-cadherin molecules. In premalignant lesions of skin tumours, P-cadherin and α6β4-integrin are overexpressed and the expression of E-cadherin is down-regulated [82], whereas in prostate cancer and in oral squamous cell carcinoma, E-cadherin and β4-integrin are markedly loss in primary and metastatic lesions, although the mechanistic pathways were not explored [83, 84].

Moreover, in ovarian cancer, P-cadherin is required for the maturation of β1 integrin in a mechanism that depends on p120ctn expression, leading to the subsequent promotion of peritoneal adhesion of metastatic cells [85]. In fact, P-cadherin and β1 integrin are induced, together with α2 and α5 integrins, by the prometastatic gonadotropin-releasing hormone (GnRH) leading to the preferential adhesion of ovarian carcinoma cells to collagen type-I and laminin. In this context, P-cadherin inhibition using RNAi leads to a decrease in cancer cell peritoneal attachment and in the number of metastatic ovarian cancer foci, suggesting that P-cadherin is involved in the early stages of metastatic cell adhesion [70].

It was also demonstrated that P-cadherin activates migration and invasion of ovarian cancer cells through IGF-1R. Cheung and colleagues found that the GnRH metastatic behaviour was shown to depend on P-cadherin expression, which in turn controls the activation of IGF-1R and the phosphorylation of p120ctn and its cytoplasmic localization [86]. P-cadherin and p-IGF-1R were significantly co-expressed in peritoneal metastatic lesions compared with paired primary ovarian tumours and, in ovarian cancer cells; GnRH induces the recruitment and formation of an IGF-1R/P-cadherin complex. P-cadherin regulation of p120ctn is IGF-1R-dependent, since the silencing of IGF-1R no longer caused GnRH-induced phosphorylation of p120ctn in P-cadherin overexpressing cells [86]. Furthermore, the same group showed that GnRH is an inducer of E- to P-cadherin switching, which is a crucial step during ovarian tumorigenesis, particularly in metastasis [80].

Human breast tumours that depend of nitric oxide (NO) oncogenic signalling are linked to poor patient survival and P-cadherin overexpression. Switzer and colleagues demonstrated that P-cadherin is a NO-inducible gene in ER-negative breast cancer cells. This mechanism involves the activation of Ras/MEK/ERK signalling pathway by NO, which in turn leads to the activation of Ets-1, a transcription factor that is implicated in metastasis, tumour progression and causes P-cadherin upregulation [87]. Furthermore, it was shown that NO treatment led to decreased E-cadherin and decreased cell adhesion indicating that NO signaling may be involved in a EMT of ER-negative breast cancer cells [88].

The Wnt/βctn signalling pathway is a known pathway associated with cadherin molecules [89] and the Wnt/βctn oncogenic effects derived from P-cadherin aberrant expression have been reported in colon cancer cells. P-cadherin knock-down in these cells reduced cell motility and proliferation, as well as decreasing cancer cell growth, invasion and liver metastasis in nude mice. These effects were associated with a reduction of βctn expression and Wnt responsive genes, including c-myc and survivin *in vitro*. This phenotype was also associated with a cadherin switch, since P-cadherin inhibition was accompanied by E-cadherin up-regulation [71].

Elevated levels of P-cadherin that occur in early oral tumour development play a critical role in neoplastic signalling networks. The ligand-dependent signalling of IGF-1R and EGFR is potentiated by P-cadherin in cell models or early tumour development. In fact, P-cadherin prolongs the activation of the mitogen-activated protein (MAP) kinase in malignant and dysplastic cells and increases the magnitude of AKT phosphorylation in dysplastic cells. P-cadherin overexpression alone is sufficient to increase steady-state levels of the mesenchymal transcription factor Snail and increase cell motility in dysplastic cells [90].

In the rabdomyosarcoma context, P-cadherin is necessary for the migratory and invasive potential of alveolar cells. In these cells, P-cadherin was shown to be a direct transcriptional target of both Pax3 and Pax3/7-FOXO1A. Further, P-cadherin overexpression in myoblasts led to a reduction in the expression and mislocalization of the classical muscle cadherins, N- and M- cadherins, thus favouring a cadherin switching, which is a hallmark of metastatic progression [91].

In the progression of melanoma, recent evidence support that the metastatic malignant melanocytes down-regulate P-cadherin expression and acquire a migratory phenotype as the result of SLUG expression, a melanocyte lineage transcription factor that was shown to downregulate this junctional protein. Furthermore, a role for SPARC in the activation of PI3K/Akt signalling, which in turn activates SLUG and predisposes to melanoma metastasis was shown in this model [92].

## P-cadherin and cancer stem cell activation

Studies show that P-cadherin contributes to the survival of aggressive cancer cells. In light of the cancer stem cell (CSC) hypothesis, these cells often exhibit stem like properties, which allow them to resist to standard cancer therapies causing tumour relapses and metastasis in cancer patients [93]. Our group has shown that P-cadherin is directly associated with the expression of the breast stem cell markers CD44, CD49f and the activity of aldehyde dehydrogenase in human breast carcinomas and in a series of breast cancer cell lines. Moreover, cell populations depleted for P-cadherin expression showed decreased *in vitro* self-renewal capacity, lower ability to grow colonies in 3D matrigel cultures and reduced tumourigenicity in nude mice. Importantly, an association was found between P-cadherin and the luminal progenitor phenotype of the normal breast differentiation hierarchy: CD49f + CD24+ [17]. Furthermore, we showed that P-cadherin is essential for the adhesion of cancer cells to extracellular matrix substrates that are critical for metastatic dissemination, namely laminin, vitronectin and fibronectin. Interestingly, the α6β4 integrin heterodimer was implicated in stem cell activity and signalling cascade downstream of P-cadherin in response to laminin, namely in the activation of FAK and Src [16]. Although the role of CSCs and P-cadherin in the metastasis needs to be further clarified, we believe that P-cadherin, as well as α6 and β4 integrin receptors, possibly in combination with other CSC markers, could be explored to better define the CSC phenotype of breast carcinomas or other malignant tumours and their corresponding metastasis.

An important link was established between P-cadherin and CSC metabolism. CSCs are hypoxia-resistant and present a preponderant glycolytic metabolism. We have demonstrated that aberrant P-cadherin expression is associated with the hypoxic/glycolytic and acid-resistant phenotype in invasive breast carcinomas, represented by a panel of markers including HIF-1α, GLUT1, CAIX, MCT1 and CD147. *In vitro*, HIF-1α stabilization was accompanied by increased membrane expression of P-cadherin and P-cadherin silencing led to a decrease of the mRNA levels of GLUT1 and CAIX and decreased mammosphere-forming efficiency [94]. P-cadherin expression shifts the metabolic program of these cells, being responsible for tumour aggressiveness and cell survival. This study was performed in a cellular context that was E-cadherin positive, but the contribution of E-cadherin in this signalling response was not explored.

A number of hypotheses have been proposed to justify the anomalous expression of P-cadherin by breast CSCs, namely, the oncofetal properties of P-cadherin protein [54], its histogenetic origin in cap cells [19] or the acquisition of a stem cell like phenotype [17]. However, although P-cadherin positive carcinomas seem to have a myoepithelial/basal-like transcriptomic programme, which points to a putative role in the cancer stem cell niche, it remains unclear which molecular mechanisms lead to the activation of P-cadherin during epithelial transformation. Several studies identify the stem cell transcription factors βctn, C/EBP-β and p63 as able to induce P-cadherin expression [95–97]. We showed that P-cadherin is expressed in cells displaying the luminal progenitor phenotype of the normal breast [17] and, notably, *BRCA1* mutation carriers exhibit an increased luminal progenitor cell population [98]. In fact, the *BRCA-1* gene is a well-known inducer of luminal cell differentiation [99] and a transcriptional repressor of basal-like genes, including P-cadherin [100]. It is still unclear whether the overexpression of P-cadherin in the *BRCA-1* inactivated basal-like breast carcinomas is related to the transformation of the luminal progenitor, which is the cell-of-origin that has been described for this malignancy [101, 102].

The metastatic dissemination of cancer cells relies on signalling pathways that are often activated in CSCs self-renewal [93], specifically the Wnt, Notch and Hedgehog pathways. The Wnt signalling pathway which is also responsible for the pluripotency of mammary stem cells [103], was found to be activated and correlated with P-cadherin expression in colon cancer cells, as mentioned previously [71]. The possible crosstalk between P-cadherin, notch and hedgehog signalling remains to be elucidated in tumours, as well as in metastasis.

## P-cadherin: is it a putative therapeutic target in cancer?

Targeting P-cadherin in cancer may be a good therapeutic approach, since normal tissues usually express very low levels of this cadherin [9]. Patients with primary tumours that overexpress P-cadherin are potential candidates for therapy regimens targeting this molecule. For this therapy to succeed, P-cadherin aggressive signalling pathways, which can be context-specific, must be scrutinized. Importantly, some studies have already pointed to a direct inactivation of P-cadherin as a therapeutic approach. Imai and colleagues reported a cancer immunotherapy study using cytotoxic T cells specific to P-cadherin peptides exhibiting anti-tumour growth effects. The authors proposed that this could provide and effective anticancer approach for pancreatic, gastric and colorectal cancers [9]. Endo and colleagues showed that one anti-P-cadherin mouse monoclonal antibody used in radioimmunotherapy significantly suppressed lung cancer growth [104]. In fact, a novel and highly selective human monoclonal antibody against P-cadherin (PF-03732010, Pfizer) has demonstrated significant anti-tumour and anti-metastatic activities in distinct P-cadherin-overexpressing tumour models, which included breast, gastric, lung, prostate and colon carcinomas, without introducing any adverse secondary effects in mice [105, 106]. PF-03732010 also reduced lymph node metastases and lowered the levels of circulating tumour cells in whole blood of P-cadherin-positive tumour bearing mice. The anti-metastatic property of the antibody was remarkable, since it significantly inhibited tumour cell infiltration into the lungs [106]. In an attempt to reach the full potential for clinical development of the antibody, PF-03732010 has completed a phase I clinical trial. The report from this first clinical trial [107] shows that, in spite of the anti-tumour effects of PF-03732010 in animal models, it was not possible to observe any clear beneficial effect in humans. However, because the participants did not experience any toxicity with the antibody, the effective therapeutic dose and its toxicity were undetermined. A new planning of the pharmacokinetic and pharmacodynamic studies in humans treated with antibodies against P-cadherin, with stratification for the different carcinomas, should translate in clearer results. Recent studies from our group also point to the potential use of the anti-cancer protein azurin in P-cadherin overexpressing breast cancer models. Azurin is a bacterial peptide secreted by *Pseudomonas aeruginosa* that leads to a decrease in the expression level of P-cadherin its invasive effects and oncogenic signalling [108, 109]. A phase I clinical trial showed evidence of azurin anti-tumour activity in human neoplasias [110].

## Conclusion

From the studies mentioned in this review, it is clear that despite P-cadherin being a classical adhesion molecule with regulatory functions in the normal context, there is a significant association between the overexpression of this molecule and poor prognosis in the carcinomas of the breast, prostate, ovary, colon and stomach. The major signalling pathways downstream of P-cadherin are context specific and include the crosstalk with α6β4 integrin, Src, Wnt, EGFR, and the interaction with other cadherin family members. Clarifying the P-cadherin signalling networks, as well as dissecting novel oncogenic pathways, is fundamental to uncover potential therapeutic targets that will counteract the effects of this protein in the invasion and the metastatic dissemination.

## Abbreviations

βctn: β-catenin
CSC: Cancer stem cell
EEM: Ectodermal dysplasia, ectrodactyly, and macular dystrophy
EGFR: Epidermal growth factor receptor
GnRH: Gonadotropin-releasing hormone
GnRHr: Gonadotropin-releasing hormone receptor
HJMD: Hypotrichosis with juvenile macular dystrophy
IGF-1R: Insulin-like growth factor receptor
MAP: Mitogen-activated protein
MMP: Matrix metalloproteinase
NO: Nitric oxide
OSCC: Oral squamous cell carcinoma
p120ctn: p120-catenin
